# Impact of the teach-back method on caregiver outcomes using the “Timing it Right” framework for hemodialysis patients

**DOI:** 10.3389/fpubh.2023.1123006

**Published:** 2023-06-23

**Authors:** Jing Huang, Xiutian Lin, Dailan Xiong, Kaiwei Huang, Xiaofei Luo, Qinwen Lin, Min Li, Ping Zhang

**Affiliations:** ^1^Guangdong Provincial People’s Hospital (Guangdong Academy of Medical Sciences), Southern Medical University, Guangzhou, China; ^2^School of Nursing, Southern Medical University, Guangzhou, China

**Keywords:** caregiver, hemodialysis, care ability, quality of life, health education

## Abstract

**Background:**

The caregivers play vital roles in the health care of hemodialysis patients. Ineffective education strategy for the caregivers negatively affects the care ability of caregivers. This study aimed to evaluate the effectiveness of the teach-back method based on the “Timing it Right” framework on the caregivers’ care ability, emotions and health-related quality of life for hemodialysis patients.

**Methods:**

The study involved 78 caregivers of 78 hemodialysis patients. Participants in the control group received routine nursing care and traditional oral health education, while those in the intervention group received health education through the teach-back method based on the “Timing it Right” framework. All participants were followed for 6 months. The degree of anxiety and depression of caregivers was evaluated through the Self-rating Anxiety Scale (SAS) and Self-Rating Depression Scale (SDS), respectively. The care ability of caregivers was assessed by the Family Caregiver Task Inventory (FCTI). The health-related quality of life of hemodialysis patients was evaluated using the 36-item Short Form Health Survey (SF-36).

**Results:**

Compared to baseline (T0), the SAS, SDS and FCTI scores of the intervention group were significantly reduced at the time of discharge (T1), three (T2) and 6 months (T3) (all *p* < 0.001). Besides, at T1, T2, and T3, the FCTI scores of the intervention group were significantly lower than that in control group (all *p* < 0.001). The SAS and SDS scores were also significantly lower in the intervention group at T1, T2, and T3 compared to the control group (all *p* < 0.001). For SF-36 scores, all domains of the intervention group were significantly higher than those of the control group at T1, T2 and T3, including physical functioning (*p* < 0.001), role physical (*p* = 0.007), bodily pain (*p* < 0.001), general health (*p* = 0.002), vitality (*p* = 0.043), social functioning (*p* = 0.016), role emotional (*p* = 0.002), and mental health(*p* = 0.025).

**Conclusion:**

The application of teach-back method based on the “Timing it Right” framework could obviously alleviate the anxiety and depression of caregivers for hemodialysis patients. Furthermore, it could significantly improve the care ability of caregivers and the quality of life of patients.

## 1. Introduction

End-stage renal disease (ESRD) is a terminal illness with increasing incidence, which affects approximately 47 million people in the United States (1, 2). The most common treatment for ESRD is hemodialysis, chosen by almost 90% of the incident patients (3, 4). Although hemodialysis can correct the electrolyte disturbance and prevent the death of patients with ESRD, it may also cause a number of complications, including malnutrition, sleep disturbances, renal anemia, renal osteopathy, and so on (5). Besides, frequent hemodialysis and restrictive diets may change the lifestyles of patients and severely affect their daily life (6). As a consequence, almost all hemodialysis patients need caregivers to take care of their life and help them manage the disease (7). The caregivers are usually the spouses, partners, adult children, parents, or other relatives of patients (8). They fulfill the role of caregivers out of love, respect, commitment, and a sense of responsibility, without receiving remuneration. Many studies suggested that the caregivers of hemodialysis patients were under high levels of care burden (9–11). High care burden would cause anxiety and depression among caregivers. Besides, the care ability of caregivers may directly affect the therapeutic effect and quality of life of hemodialysis patients (12). Therefore, the emotion and care ability of caregivers for hemodialysis patients are worthy of special attention. Unfortunately, the caregivers are always the group being ignored. Few studies have investigated how to improve the mood and care ability of caregivers for hemodialysis patients (13).

Lack of knowledge about hemodialysis and related nursing care is the main factor inducing anxiety and depression in caregivers (14). This also directly affects the quality of care and even the survival rate of hemodialysis patients (15). Professional health education can improve the care ability of caregivers and decrease their confusion. Nonetheless, a report indicated that people would instantly forget 40 to 80% of the medical information presented to them (16). Therefore, it is vital to implement effective education strategies for clinicians, patients, and their caregivers. One of the effective education strategies is the teach-back method (17). In this method, the participants are requested to recount their understanding in their own words after receiving health education (18). Through this method, the clinicians can provide individual recommendations to participants in time according to their learning capacity (19). Previous studies have confirmed the effectiveness of the teach-back method in improving patient-clinician communication and the self-management of patients (20–22). At various stages of the disease process, both the patient and caregiver may experience different types of stress and emotions. The characteristics of the patient’s condition in each period should be fully mastered, taking into account the needs of emotion, information, and evaluation. In 2008, Cameron et al. (23) proposed the “Timing It Right” Framework for addressing the support needs of family caregivers to the patients in different phases of disease. As the theoretical basis of continuous nursing, “Timing It Right” Framework splits the disease process into five phases, including diagnostic, stationary, preparation, implementation, and adaptability, as the theoretical foundation for continuous nursing (23, 24). This framework can assist patients in adjusting the family and social environment as quickly as possible, improve the self-management and adherence of patients, hasten the healing process of patients, and offer research opportunities and theoretical support for the ongoing nursing of chronic diseases (25, 26). In this study, we focused on the caregivers of hemodialysis patients. The aim was to evaluate the utility and efficacy of this teach-back method based on the “Timing it Right” framework on the care ability and emotions of caregivers and the quality of life of patients.

## 2. Materials and methods

This was a non-randomized control study. Seventy-eight hemodialysis patients and their 78 primary caregivers were included in the current study. All patients received hemodialysis in Guangdong Provincial People’s Hospital between November 1, 2021, and May 31, 2022. The inclusion criteria of hemodialysis patients were: (1) age ≥ 18 years, (2) newly diagnosed end-stage kidney disease (ESRD), with glomerular filtration rate (GFR) less than 15 mL/(min·1.73 m^2^), (3) underwent regular hemodialysis, and (4) the score of Care Dependency Scale for Rehabilitation (CDS-R) was less than or equal to 68. The exclusion criteria were (1) received temporary hemodialysis, (2) the presence of serious complications, such as heart failure or serious infection, and (3) psychiatric disorders or cognitive illnesses. Patients and caregivers were divided into intervention group and control group using a quasi-randomization method. They were allocated to either the intervention or control group based on the order in which they were recruited into the study.

Each patient was asked to identify his or her primary caregiver. The primary caregiver should fulfill the following criteria: (1) age ≥ 18 years, (2) accompanied patient during the whole hemodialysis process, (3) responsible for the patient’s daily life. The exclusion criteria for the caregiver were (1) provided care for remuneration, (2) psychiatric disorders or cognitive illnesses, and (3) hearing or visual impairment.

Both patients and caregivers were informed of the study objective and data confidentiality. Written informed consents were obtained from patients and their primary caregivers prior to participating in this research. This study was approved by the Ethics Committee of Guangdong Provincial People’s Hospital and was performed in accordance with the Code of Ethics of the World Medical Association (Declaration of Helsinki).

Caregivers were asked to provide their sociodemographic information including age, gender, marital status, educational level, occupation, annual income, relationship with patient, whether he or she lived with the patient, and their health statuses.

Self-rating Anxiety Scale (SAS), developed by Zung in 1971, was used to assess the anxiety of caregivers (27). This is a self-administered scale comprised of 20 questions. The caregivers scored each item on a 4-point Likert scale ranging from 1 to 4, according to the frequency of symptoms over the past week. The standard score is equal to the total raw score multiplied by 1.25. The standard score is classified into four categories, which are “no anxiety” (25–49), “minimal to mild anxiety” (50–59), “moderate to marked anxiety” (60–69), and “severe anxiety” (>70).

Self-rating Depression Scale (SDS), with 20 items, was used to evaluate the severity of depressive symptom in caregivers (28). There are 10 positively worded and 10 negatively worded questions. Each question is scored ranging from 1 (a little of the time) to 4 (most of the time). The standard score is also equal to the total raw score multiplied by 1.25. The total score ranges from 25 to 100, classified as ‘normal range’ (25–49), “mildly depressed” (50–59), “moderately depressed” (60–69), and “severely depressed” (>70).

In order to evaluate the care ability of caregivers comprehensively, the Family Caregiver Task Inventory (FCTI) was used in this study. This scale consists of 25 items including 5 dimensions. Each entry adopts 3-point Likert scoring method: 0-point means not difficult, 1-point means difficult, and 2-point means extremely difficult. The total score of this scale is 50 points. A higher score reflects more difficulty and worse care ability.

For hemodialysis patients, the basic sociodemographic data were collected, including age, gender and the frequency of hemodialysis per week. The Care Dependency Scale for Rehabilitation (CDS-R) is a short assessment instrument that measures the care dependency of patients regarding physical and psychosocial aspects. It is used to assess changes in the degree of dependency from dependent to independent, which is essential in rehabilitation (29). The patients were also asked to fill in CDS-R, in order to evaluate their care dependency regarding to the physical and psychosocial aspects.

The health-related quality-of-life of hemodialysis patients was measured by the 36-item Short Form Health Survey (SF-36) (30). This is a questionnaire consisting of 36 questions and categorized into 8 health domains: physical functioning, role physical, bodily pain, general health, vitality, social functioning, role emotional, and mental health. For each item, different options have different score weightings and the final score ranges from 0 (worst general health status) to 100 (best health status). A higher score indicates better quality of life.

Both participants in the intervention group and control group received the initial evaluation before the first hemodialysis right after the diagnosis of ESRD. Three questionnaires in Chinese, including SAS, SDS and FCTI, were used to assess the degree of anxiety, depression and care ability of caregivers. The initial health-related quality of life of hemodialysis patients was evaluated by SF-36 in Chinese. The time for initial evaluation was regarded as the time of study entry (T0). The hemodialysis patients and their primary caregivers in the control group received routine nursing care and traditional oral health education while in hospital.

The knowledge about hemodialysis and relevant nursing care was presented to the primary caregivers as texts, videos and pictures while the patients were admitted to hospital, at the time of discharge, 1 month and 3 months after the first hemodialysis (Supplementary File S1). In addition to the conventional nursing care, the participants in the intervention group received the health education through teach-back method based on the “Timing it Right” framework. A trained nurse was responsible for conducting the health education in the office when patients and caregivers were in the hospital. In addition, close communication and health education was established between the investigators and caregivers through cellphone after discharge. The health information related to the patients and hemodialysis was provided and explained to the patients and their caregivers. Then, the caregivers were asked to recount their understanding in their own words. Further individual professional guidance was introduced to each caregiver according to their understanding and learning capacity. The investigators could solve the caregivers’ questions and corrected their improper procedures in time.

All caregivers were evaluated by SAS, SDS and FCTI at the time of discharge (T1). All participants were followed for half years. The caregivers were reassessed at 3 months (T2) and 6 months (T3) by SAS, SDS and FCTI. The health-related quality of life of hemodialysis patients was assessed by SF-36 at T3. Two researchers (JH and XTL) were responsible for the data collection.

### 2.1. Statistical analysis

All statistical analyzes were performed by SPSS software version 25.0 (IBM Corporation; United States). The normality of data was checked using graphical methods, which was quantile-quantile plot. The general characteristics of the participants were analyzed by Student’s t test or Pearson’s *χ*^2^ test. The Mann–Whitney U test was used to compare the frequency of hemodialysis per week between the patients in intervention group and control group. The difference of SAS, SDS and FCTI scores between intervention and control group was determined by Two-way mixed ANOVA. The variation trends of SAS, SDS and FCTI scores from T0 to T3 were detected using One-way repeated measures ANOVA test. The variation of SF-36 scores between two groups at T0 and T3 was determined by Wilcoxon signed-rank test. A threshold of p less than 0.05 was considered statistically significant.

## 3. Results

### 3.1. General characteristics

Thirty-nine hemodialysis patients and their primary caregivers were included in the intervention group and control group, respectively. The general characteristics of the patients and their caregivers in the two groups were described in Table 1. There were no significant differences between two groups in the age and gender of patients and caregivers (all *p* > 0.05). The marital statuses, educational levels, occupations, and annual incomes of caregivers showed no statistically significant difference between two groups. The most common relationship between caregiver and patient was partner, and the difference was not statistically significant (*p* = 0.287). No difference was observed in the frequency of hemodialysis per week for patients between two groups (*p* = 0.303).

**Table 1:** General characteristics of intervention group and control group.

	Intervention group (*n* = 33)	Control group (*n* = 33)	*p* value
Age (y), M ± SD			
Care giver	44.36 ± 13.88	44.87 ± 15.71	0.879^a^
Patient	54.87 ± 11.19	59.38 ± 11.92	0.089^a^
Gender of caregiver			
Male	15 (38.46%)	12 (30.77%)	0.475^b^
Female	24 (61.54%)	27 (69.23%)	
Gender of patient			
Male	29 (74.36%)	25 (64.10%)	0.326^b^
Female	10 (25.64%)	14 (35.90%)	
Relation between caregiver and patient, *n* (%)			
Partner	19 (48.72%)	19 (48.72%)	0.287^b^
Child	14 (35.90%)	18 (46.15%)	
Other	6 (15.38%)	2 (5.13%)	
Marital status of caregiver, *n* (%)			
Unmarried	6 (15.38%)	8 (20.51%)	0.803^b^
Married	14 (35.90%)	12 (30.77%)	
Other	19 (48.72%)	19 (48.72%)	
Educational level of caregiver, *n* (%)			
High school or lower	28 (71.79%)	29 (74.36%)	0.799^b^
University degree or higher	11 (28.21%)	10 (25.64%)	
Occupation of caregiver, *n* (%)			
Yes	33 (84.62%)	28 (71.79%)	0.170^b^
No	6 (15.38%)	11 (28.21%)	
Annual income of caregiver (Yuan), *n* (%)			
Less than 50 k	11 (28.21%)	10 (25.64%)	0.868^b^
50 k ~ 100 k	16 (41.02%)	15 (38.46%)	
100 k ~ 200 k	7 (17.95%)	10 (25.64%)	
More than 200 k	5 (12.82%)	4 (10.26%)	
Times of hemodialysis per week for patient, *n* (%)			
Twice	12 (30.77%)	8 (20.51%)	0.303^c^
Thrice	27 (69.23%)	31 (79.49%)	

a Student’s *t*-test.

b Pearson’s *χ*^2^ test.

c Mann–Whitney U test.

M ± SD, mean ± standard deviation.

### 3.2. The degree of anxiety and depression among caregivers

**Figure 1:**
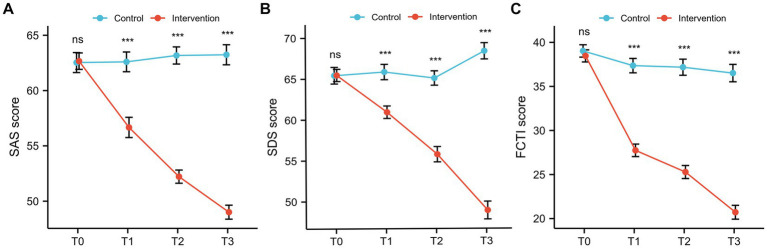
The SAS, SDS, FCTI scores for caregivers. **(A)** SAS scores; **(B)** SDS scores; **(C)** FCTI scores.

In the initial phase (T0), the SAS scores were 62.66 ± 4.64 in the intervention group and 62.52 ± 5.64 in the control group (*p* = 0.913). For SDS scores at T0, the intervention group was 65.45 ± 4.66, while the control group was 65.42 ± 6.33 (*p* = 0.98) (Figures 1A,B; Table 2). This indicated that all caregivers in both groups presented the moderate degree of anxiety and depression at the beginning. After receiving health education through teach-back method based on the “Timing it Right” framework, both SAS scores and SDS scores in the intervention group showed the significant downward trends over time (both *p* < 0.001) (Table 2). Besides, the SAS and SDS scores in the intervention group were significantly lower than those in the control group at T1, T2, and T3 (all *p* < 0.001) (Figures 1A,B).

**Table 2:** The SAS, SDS, FCTI scores for caregivers.

	T0	T1	T2	T3	*p* value^a^
SAS scores					
Control group	62.53 ± 5.64	62.60 ± 5.56	63.17 ± 4.85	63.28 ± 5.66	0.741
Intervention group	62.66 ± 4.64	56.67 ± 5.72	52.22 ± 3.73	49.01 ± 3.94	<0.001
SDS scores					
Control group	65.42 ± 6.33	65.83 ± 5.86	65.06 ± 5.49	68.35 ± 6.20	0.029
Intervention group	65.45 ± 4.66	60.92 ± 4.77	55.75 ± 5.85	48.89 ± 6.79	<0.001
FCTI scores					
Control group	39.03 ± 4.39	37.36 ± 5.10	37.18 ± 5.71	36.51 ± 6.17	0.085
Intervention group	38.46 ± 4.28	27.74 ± 4.44	25.28 ± 4.62	20.72 ± 4.92	<0.001

a One-way repeated measures ANOVA test.

### 3.3. The care ability of caregivers

The FCTI was used to assess the care ability of caregivers, which was shown in Figure 1C and Table 2. Compared to baseline (T0), the FCTI scores of the intervention group decreased significantly at T1, T2, and T3 (*p* < 0.001). Furthermore, at T1, T2, and T3, the FCTI scores in the intervention group were significantly lower than that in the control group (all *p* < 0.001), while no difference was observed at baseline (T0) (*p* > 0.05).

### 3.4. The health-related quality of life of hemodialysis patients

**Figure 2:**
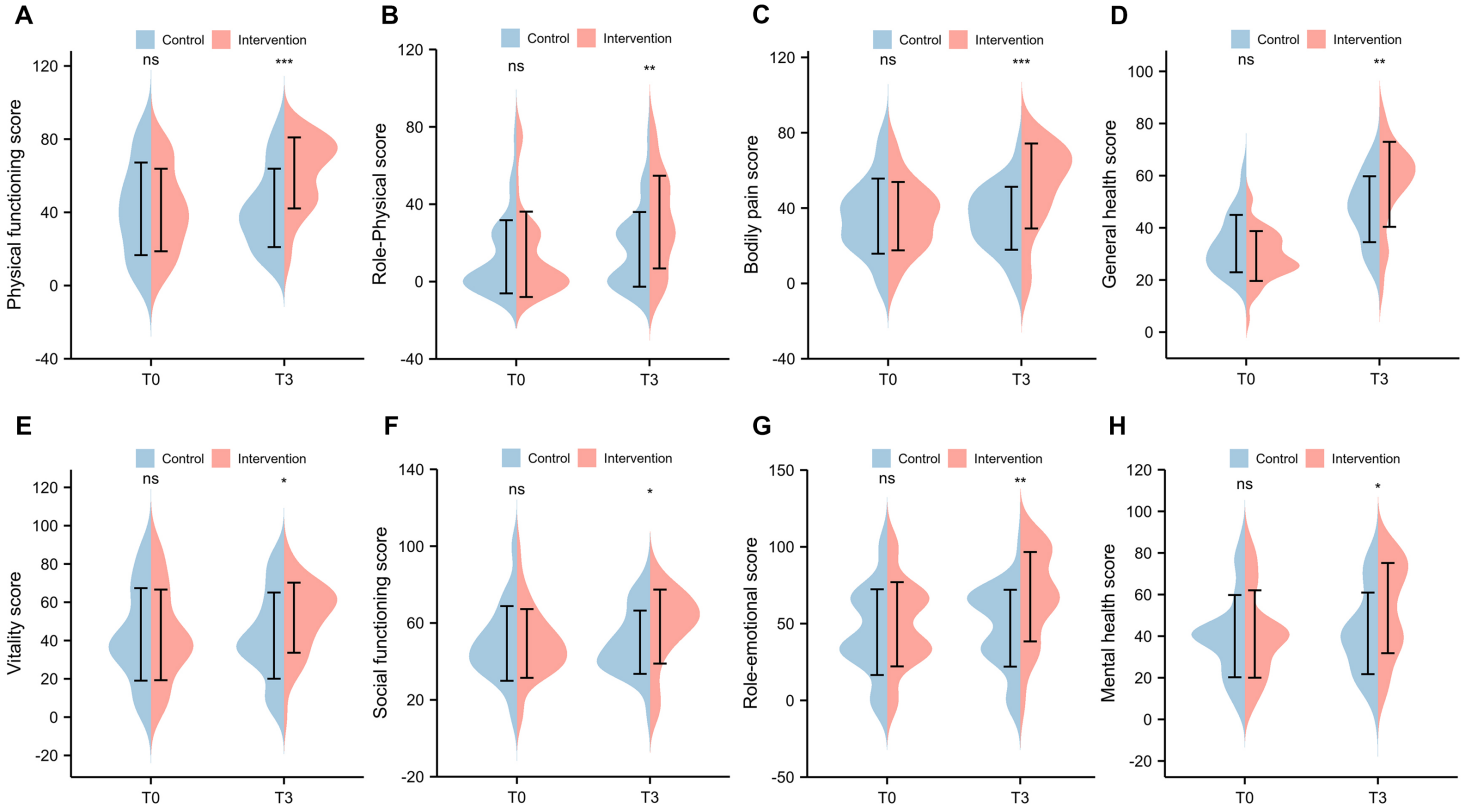
The SF-36 scale scores for hemodialysis patients. **(A)** Physical functioning; **(B)** Role physical; **(C)** Bodily pain; **(D)** General health; **(E)** Vitality; **(F)** Social functioning; **(G)** Role emotional; **(H)** Mental health.

The SF-36 scores at baseline (T0) and half-year follow-up (T3) were shown in Figure 2. Compared to control group, the SF-36 scores of the intervention group at T3 were significantly increased in all health domains, including physical functioning (*p* < 0.001), role physical (*p* = 0.007), bodily pain (p < 0.001), general health (*p* = 0.002), vitality (*p* = 0.043), social functioning (*p* = 0.016), role emotional (*p* = 0.002), and mental health (*p* = 0.025). No difference in the SF-36 scores was observed between two groups at T0 (all *p* > 0.05).

## 4. Discussion

Long-term hemodialysis would negatively affect the physical, psychological, and socioeconomic aspects of patients’ lives, which results in their dependence on caregivers (31, 32). Caregivers are people mostly involved the care of patients and help them to adapt and manage their chronic disease during the course of illness and treatment (33). Our study aimed to evaluate the effectiveness of the teach-back method, based on the “Timing it Right” framework, in improving the care ability, emotions, and health-related quality of life of caregivers for hemodialysis patients. We found that the intervention group showed significant improvements in SAS, SDS, and FCTI scores at the time of discharge, 3 months, and 6 months compared to baseline. The intervention group also had significantly lower FCTI, SAS, and SDS scores compared to the control group at all time points. Additionally, all domains of the SF-36 scores were significantly higher in the intervention group compared to the control group at all time points.

Our results suggested that the teach-back method could dramatically improve the care ability of caregivers and relieved their anxiety and depression. The corner stone for improving the patients’ self-management capacity and caregivers’ care ability is to help them understand the disease and remember the medical advices at various stages of the illness and management (34). However, a variety of factors, including redundant medical information, low literacy, and inappropriate communication methods, lead to inefficient patient-clinician communication (35). Previous research showed that the patients could only comprehend and retain less than half of the medical information presented to them (36). One study reported that 75% of doctors believed health education went well with their patients, but only 21% of patients reported satisfactory outcomes (37). Ineffective education strategy and communication may lead to drug misuse, complications, and poor therapeutic effect, and so on (38). Meanwhile, confusion about the medical information may cause the anxiety and depression in patients and their caregivers. Therefore, it is necessary to implanting better methods to improve effectiveness of health education at the patient-clinician interface.

As a simple educational strategy, teach-back method, was advocated to use in chronic disease education. A number of studies have verified that the use of teach-back method could improve the patients’ comprehension and informed consent, in comparison to traditional communication modes (39, 40). Griffey et al. performed a randomized controlled trial and found that teach-back method helped emergency patients receive more medical knowledge and gain better outcome, compared to the standard discharge instructions (41). A systematic review with 20 studies also confirmed the effectiveness of the teach-back method across a wide range of settings, populations and outcome measures (42). Therefore, we should provide effective health education to the caregivers and patients.

The results of our study also indicated that the teach-back method based on the “Timing it Right” framework could significantly improve the care ability of the caregivers of hemodialysis patients, and consequently improved the patients’ quality of life. Patients plan to start their long-term repeated hemodialysis needs to adapt to the various status of physical and psychological changes at different stages. In addition, their caregivers are also required to meet the exact needs of the patients at different stages of the hemodialysis process. The advantage of this framework is that it can help health care professionals to provide more timely and appropriate support to caregivers by recognizing their phase-specific needs for information, education, training, and emotional support. As a consequence, this teach-back method based on the “Timing it Right” framework is worth popularizing and applying in routine patient-clinician communication. In addition, our study found that the patients’ quality of life was better in the intervention group than those in the control group at 6-month. This further demonstrated the teach-back method based on the “Timing it Right” framework could improve the care ability of caregivers.

Compared to other studies, the strength of our study is that we focused on the caregivers of hemodialysis patients. This study was the first to explore the value of the teach-back method based on the “Timing it Right” framework in improving the care ability and emotions of these caregivers. However, a few limitations cannot be ignored. Firstly, our research was a non-randomized controlled study and it was not double-blinded. It may cause selection bias, information bias and confounding bias. Secondly, a multicenter study with larger sample volumes is needed to assess the value of this teach-back method. Last but not least, the subsequent changes in emotion and care ability of the caregivers over a longer follow-up period should be evaluated.

## 5. Conclusion

In conclusion, this study revealed that the application of teach-back method based on the “Timing it Right” framework could obviously alleviate the anxiety and depression of caregivers for hemodialysis patients. Most importantly, it could significantly improve the care ability of caregivers and the quality of life of patients. These findings provide the evidence to support the application of teach-back method based on the “Timing it Right” framework in patient-clinician communication.

## Data availability statement

The raw data supporting the conclusions of this article will be made available by the authors, without undue reservation.

## Ethics statement

The studies involving human participants were reviewed and approved by Guangdong Academy of Medical Sciences. The patients/participants provided their written informed consent to participate in this study.

## Author contributions

JH, XLi, DX, KH, XLu, QL, ML, and PZ contributed to the study conception and design. Study design and project development was performed by PZ. Data collection and analysis was performed by JH, XLi, DX, KH, XLu, QL, and ML. The first draft of the manuscript was written by JH and PZ. All authors contributed to the article and approved the submitted version.

## Funding

This research was funded by Nursing Scientific Research Foundation of Guangdong Provincial People’s Hospital, Guangdong Academy of Medical Sciences, Grant number DFJH2021015.

## Conflict of interest

The authors declare that the research was conducted in the absence of any commercial or financial relationships that could be construed as a potential conflict of interest.

## Publisher’s note

All claims expressed in this article are solely those of the authors and do not necessarily represent those of their affiliated organizations, or those of the publisher, the editors and the reviewers. Any product that may be evaluated in this article, or claim that may be made by its manufacturer, is not guaranteed or endorsed by the publisher.
