# Supplementary material for: Impact of the teach-back method on caregiver outcomes using the “Timing it Right” framework for hemodialysis patients
Source: Front Public Health. 2023 Jun 23;11:1123006. doi: 10.3389/fpubh.2023.1123006 (PMC10326316; doi:10.3389/fpubh.2023.1123006)
Supplement: Supplementary file 1 [file Table_1.docx]

**Appendix 1**

**Part 1**

**Questionnaire on the older people’s demand for home- and community-based integrated care**

ID：＿＿ Community Name： ＿＿＿＿＿＿ Phone Number：＿＿＿＿＿＿

Guideline: The following questions are designed to find out your level of demand for integrated health care services. We have classified the intensity of demand as 5="very needed", 4="needed", 3="occasionally needed", 2="not very needed", 1="very unneeded". Please fill in the form faithfully and tick the appropriate mark.

| Items | 5=very needed | | | 4=needed | | 3=occasionally needed | 2=not very needed | | 1=very unneeded | |
| --- | --- | --- | --- | --- | --- | --- | --- | --- | --- | --- |
| **A1 Basic life services** | | | | | | | | | | |
| preparing meals, | |  |  | |  | | |  | |  |
| housekeeping | |  |  | |  | | |  | |  |
| on-site service (home visit) | |  |  | |  | | |  | |  |
| shopping | |  |  | |  | | |  | |  |
| transportation | |  |  | |  | | |  | |  |
| hotline service | |  |  | |  | | |  | |  |
| day care | |  |  | |  | | |  | |  |
| safety | |  |  | |  | | |  | |  |
| **A2 Medical–nursing services** | | | | | | | | | | |
| disease treatment | |  |  | |  | | |  | |  |
| home sickbed care | |  |  | |  | | |  | |  |
| bidirectional referral | |  |  | |  | | |  | |  |
| preventive care | |  |  | |  | | |  | |  |
| Chinese medicine service | |  |  | |  | | |  | |  |
| injections | |  |  | |  | | |  | |  |
| **A3 Rehabilitation services** | | | | | | | | | | |
| massage | |  |  | |  | | |  | |  |
| acupuncture | |  |  | |  | | |  | |  |
| physical therapy | |  |  | |  | | |  | |  |
| rehabilitation guidance | |  |  | |  | | |  | |  |
| **A4 Psycho-spiritual support services** | | | | | | | | | | |
| spiritual solace | |  |  | |  | | |  | |  |
| psychological counseling | |  |  | |  | | |  | |  |
| company | |  |  | |  | | |  | |  |
| **A5 Health education services** | | | | | | | | | | |
| health counseling | |  |  | |  | | |  | |  |
| health guidance | |  |  | |  | | |  | |  |
| first aid training | |  |  | |  | | |  | |  |
| life skills training | |  |  | |  | | |  | |  |
| **A6 Social participation services** | | | | | | | | | | |
| provision of exercise venues and opportunities | |  |  | |  | | |  | |  |
| voluntary activities | |  |  | |  | | |  | |  |
| cultural and entertainment services | |  |  | |  | | |  | |  |
| **A7 Social aid services** | | | | | | | | | | |
| legal aid | |  |  | |  | | |  | |  |
| dispute mediation | |  |  | |  | | |  | |  |

**Part 2**

**Questionnaire on influencing factors of the older people’s demand for home- and community-based integrated care, based on extended Andersen’s behavioral model of health service utilization**

| Predisposing | Demographic | Age | 1=60-69, 2=70-79, 3=80-89, 4=90-99 |
| --- | --- | --- | --- |
|  |  | Sex | 1=Man, 2=Female |
|  |  | Marital status | 1=with spouse, 2=without spouse (including unmarried, separated, or widowed) |
|  |  | Household registration | 1=urban, 2=rural |
|  |  | Jurisdiction | 1=KaiFu, 2=FuRong, 3=YuHua, 4=YueLu, 5=TianXin, 6=JingKai |
|  | Social Structure | Pre-retirement occupation | 1＝Public institution staff, 2＝Corporate staff, 3＝Technical staff, 4＝Self-employed, 5＝Farmer, 6= Freelancer |
|  |  | Education | 1=No or little literacy, 2=Primary school, 3=Junior high school, 4=High school or technical secondary school, 5=Junior college, 6=Bachelor and above |
|  |  | Neighborhood relations | 1=very bad, 2=bad, 3=fair, 4=better, 5=very good |
|  | Health Beliefs | Knowledge of integrated care | 1=Not at all, 2=Not at all, 3=Fairly well, 4=Fairly well, 5=Very well |
|  |  | Willingness to pay for integrated care | 1=very reluctant, 2=reluctant, 3=fair, 4=more willing, 5=very willing |
| Enabling | Family | Economic situation | 1=very poor, 2=poor, 3=fair, 4=good, 5=very good |
|  |  | Disease burden | 1=very heavy, 2=heavier, 3=fair, 4=easy, 5=very easy |
|  |  | Health insurance | 1=Urban employees’ insurance，2=Urban residents’ insurance，3=New rural social endowment insurance, 4=Commercial medical insurance, 5=None |
|  |  | Living conditions | 1＝Living with children，2＝Living with spouse, 3＝Living with children and spouse, 4＝Living alone, 5=Other |
|  |  | Number of children | Continuing variables( 0，1，2，3，……) |
|  |  | Degree of child support | 1=very unsupportive, 2=not supportive, 3=fair, 4=more supportive, 5=very supportive |
|  |  | Family relationships | 1=very bad 2=not good, 3=fair, 4=better, 5=very good |
|  |  | Primary caregiver | 1=Self, 2=Spouse, 3=Children, 4=Other relatives or friends, 5=Nanny or hourly worker |
|  |  | Informal support（mainly refers to caring for children and grandchildren） | 1=Never, 2=Occasional, 3=Often |
|  | Community | Service attitude of community staff | 1=very bad, 2=not good, 3=fair, 4=better, 5=very good |
|  |  | Quality of community medical services | 1=very bad, 2=not good, 3=fair, 4=better, 5=very good |
|  |  | Infrastructural facilities | 1=very bad, 2=not good, 3=fair, 4=better, 5=very good |
|  |  | Satisfaction with resources | 1=very dissatisfied, 2=not satisfied, 3=fair, 4=satisfied, 5=very satisfied |
|  |  | Convenience of access to clinics | 1=very inconvenient, 2=not convenient, 3=fair, 4=more convenient, 5=very convenient |
| Need | Perceived | Self-assessed health status | 1=very bad, 2=not good, 3=fair, 4= better, 5=very good |
|  |  | Self-assessed mental status | 1=very bad, 2=not good, 3=fair, 4 =better, 5=very good |
|  |  | Number of chronic diseases | Continuing variables( 0，1，2，3，……) |
|  |  | Satisfaction with state of pension | 1=very dissatisfied, 2=not satisfied, 3=fair, 4=satisfied, 5=very satisfied |
|  | Evaluated | Degree of impaired capacity | 1=heavily impaired, 2=moderately impaired, 3=slightly impaired, 4=unimpaired |
| Perception of Aging | Traditional Views | Acceptability of community social care rather than family care | 1=very unacceptable, 2=not acceptable, 3=average, 4=more acceptable, 5=very acceptable |
